# Effective Coupling Model to Treat the Odd–Even Effect on the Current–Voltage Response of Saturated Linear Carbon Chains Single-Molecule Junctions

**DOI:** 10.1021/acsomega.4c00457

**Published:** 2024-08-06

**Authors:** Hugo Cabrera-Tinoco, Augusto C. L. Moreira, Renato Valencia-Bedregal, Luis Borja-Castro, Adela Perez-Carreño, Aldo Lalupu-García, Carlos Mendoza-Alejo, Crispin H. W. Barnes, Ji Won Seo, Luis De Los Santos Valladares

**Affiliations:** † Facultad de Ingenierías, Universidad Continental, Lima 15311, Peru; ‡ Núcleo Interdisciplinar em Ciências Exatas e da Natureza (NICEN), Universidade Federal de Pernambuco, 55014-900 Caruaru, Pernambuco , Brazil; § Laboratorio de Cerámicos y Nanomateriales, Facultad de Ciencias Físicas, Universidad Nacional Mayor de San Marcos, Ap. Postal 14-0149 Lima, Peru; ∥ College of Science and Technology Convergence, Yonsei University, 1 Yonseidae-gil, Wonju, Gangwon-do 26493, South Korea; ⊥ Programa de Pós-Graduação em Ciências de Materiais, Centro de Ciências Exatas e da Natureza, Universidade Federal de Pernambuco, 50670-901 Recife, Pernambuco, Brazil; # Cavendish Laboratory, Department of Physics, University of Cambridge, J. J Thomson Avenue, Cambridge CB3 0H3, U.K.

## Abstract

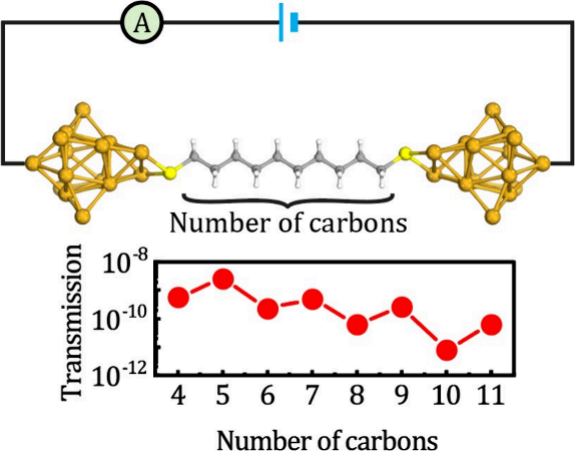

The calculation of the electrical charge transport properties of alkanes *C*_*n*_*H*_2*n*_*S*_2_ with (*n* = 4–11) was performed to understand the odd–even effect on its current–voltage response. The extended molecule and broadband limit models were used to describe the molecular junction and covalent coupling with the electrodes. It was shown that among the participating molecular orbitals, HOMO and HOMO–1 are the ones with the most charge transport contribution. Moreover, the odd–even effect is caused by the alternation of the eigenvalues of some frontier orbitals as a function of the number of carbons, especially the HOMO that dominates the electrical transport. It could also be noted that when the current is analyzed outside the resonance, the relationship with the number of carbons exponentially decays, confirming the reports in the literature. To the best of our knowledge, a first principle study of the odd–even effect in symmetric systems composed by linear saturated carbon chains covalently coupled to electrodes has not been reported yet.

## 1 Introduction

The miniaturization of electronic components is related to the increase in the capabilities and functionalities of technological devices.^1−5^ Due to this, there is a growing interest in searching for alternative materials to the top–down approach of the semiconductor-based technology. In this sense, since Aviram and Ratner^6^ suggested the idea of using an organic molecule as a rectifying diode, molecular electronics has become a discipline to which great effort has been devoted during the last decades. The low dimensionality, the reduced cost of synthesis, and the variety of physical and chemical properties are some of the advantages of using organic molecules as active components in electronics technology.^7−11^

First, it is necessary to understand the behavior of the current–voltage response of the molecular device and its main characteristics. For example, in the case of the alkanes family, it is well-known that the conductance decays exponentially with increasing size.^4,12^ Another important feature is the parity of a molecular chain component.^13,14^ Whitesides reported that there is an effect on the current density depending on the parity of the linear chains, i.e., on the number of methylene groups in the structure of the self-assembled monolayer (SAM) of an alkanethiolate molecule^15^. In recent experimental works, it was confirmed that this effect was due to a physical and chemical characteristic of the SAM, and it was not an artifact of the statistical treatment of the measurements.^16,17^ It should be noted that the measurements are focused on the study of SAMs, where the molecules were anchored by a covalent bond to an electrode (generally gold or silver) and the coupling with the other electrode by a van der Waals type interaction.^16,17^ The explanation for the odd–even effect could be associated with the geometry of the molecular region that interacts with the weakly coupled electrode, the thickness of the SAM, the change in the dipole moment of the SAM molecules, and the alternation of the frontier orbital energies.^13,18,19^

In this sense, the odd–even effect has been shown to be an important factor influencing the properties of molecular electronic devices.^20^ For example, in order to design a molecular rectifier diode, an asymmetric molecule is usually sought to generate the rectifying effect. Two proposals of this type were studied by Wei et al.^18^ and Wang et al.,^21^ where the molecular device was formed by a chain of alkanes joined to a molecule of bipyridyl and ferrocene, respectively. The computational results found that there is an odd–even effect on the current rectifying properties. The parity of the methylene chain is the cause of an oscillation feature in the rectification ratio. Wang and Wei showed that the occupied orbitals are the conduction channels that determine the transport since they are closer to the Fermi level, and the alternation of the energies of these orbitals is the reason for the odd–even effect.^18,21^ It is important to note that Wang and Wei calculations simulate transport on a single molecule, unlike other calculations, which simulate a SAM. This opens the possibility that the odd–even effect appears in single-molecule devices, and it would not be associated only with SAMs.

On the other hand, the most used theoretical tool for studying the electronic transport of an organic molecule between two metallic electrodes is the nonequilibrium Green function (NEGF) formalism, together with the density functional theory (DFT).^22−24^ The NEGF-DFT treatment has been used to describe the charge transport from a wide variety of molecular systems. However, despite its success in reproducing the experimental measurements to a large extent, the NEGF-DFT has the disadvantage of being computationally expensive. We proposed a homemade transport formalism to study the transport properties in a simpler way using complex absorbing potentials (CAPs) to model the effects of semi-infinity right and left contacts.^13,25,26^ In this article, we will focus on the study of electrical response by symmetrical systems composed of single molecules of alkanes *C*_*n*_*H*_2*n*_*S*_2_ with (*n* = 4–11) coupled to two gold electrodes via thiol groups, with the purpose of exploring the odd–even effect in these symmetric molecules at a density functional theory (DFT) level with the CAPs technique. Also, it should be noted that in dealing with symmetric systems (with symmetric coupling to the contacts), electrical current rectification is absent in our results, since asymmetry is the key feature for this effect.^18,21^

## 2 Theoretical Model and Computational Details

**Figure 1:**
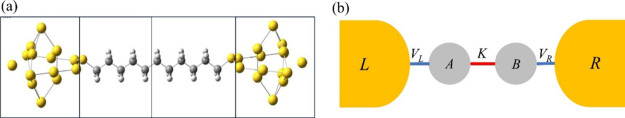
(a) Molecular junction model for undecanedithiol. (b) Schematic model for the system.

The geometry of the molecular junction in our model consists of the organic molecule coupled to two gold clusters through a thiol group, forming the so-called extended molecule,^13,27,28^ as shown in Figure 1a. Both gold clusters—each of the two gold clusters has 15 atoms—will be used to represent the interaction between the molecule and the electrodes. A total of eight systems were studied: four even alkanes (*n* = 4, 6, 8, and 10) and four odd alkanes (*n* = 5, 7, 9, and 11). The terms even and odd refer to the number of carbon atoms in the molecular structure.

The structure of the molecular junction was relaxed keeping the geometry of the gold clusters fixed.^29^ The distance between the clusters was considered as a degree of freedom to optimize. To speed up the calculations in this process, only four of the 15 gold atoms in each cluster were used. After the relaxation process, the electronic structure of each molecular junction was determined through a single point calculation. Elements such as eigenvectors and eigenvalues of molecular orbitals are necessary to implement our transport model. The optimization structure and single point calculations were performed using the GAUSSIAN 09 software.^30^ The chemical model was described by the functional hybrid B3LYP. The base set of atomic orbitals used was LANL2DZ and 6-31G(d,p) for gold atoms and organic atoms,^13,29^ respectively.

With the electronic structure in hands, the electric current through the molecule is determined by the Landauer–Büttiker eqs 4a-6, i.e.,
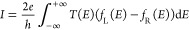
1

In eq 1, *f*_L_(*E*) and *f*_R_(*E*) are the Fermi functions of the left and right electrodes, respectively, and *T*(*E*) is the transmission function that, disregarding multiple scattering processes between the left and right contacts, can be defined as^22,31,32^
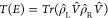
2

In eq 2, we have ρ̂_L_[ ρ̂_R_] as the left [right] density of states of the contacts, and *V̂* is a matrix that couples the left and the right sides of contacts. It should be noted that the evolution of the molecule and electrode system is determined by the Hamiltonian *Ĥ* = *Ĥ*_0_ + Σ̂^r^(*E*), where *Ĥ*_0_ is the Hamiltonian of the molecule, and Σ̂^r^(*E*) is the (retarded) self-energy that couples the molecule with the electrodes. The general form of the self-energy is given by:^23,33,34^
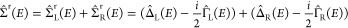
3with the real part Δ̂_L/R_(*E*) shifting the eigenvalues and the imaginary part Γ̂_R/L_(*E*) broadening them. Usually, the self-energy of eq 3 is calculated in a self-consistent manner^35,36^ through the relationship between the density matrix and the Green′s function. However, this process can be simplified using the wide band limit (WBL) approximation^28^ with a complex absorbing potential (CAP) that resembles the semi-infinity electrode^37^. In this approximation, since only the imaginary part effectively contributes, the Green’s function can be simplified, and the transmission function from eq 2 can be calculated using a simple expression, as we will show in the next section.

### 2.1 Model Self-Energy and Transmission

Once the electronic structure is available, the next step in our approach consists of modeling the self-energy. For this purpose, we assume a wide band limit approximation^28^ where the interaction with the semi-infinite substrates is described by a purely imaginary energy-independent self-energy, i.e., Δ_L/R_(*E*) = 0 and Γ_L/R_(*E*) = Γ_L/R_ = −2 Im [Σ_T/B_^r^]. The term Σ_*X*_^r^ (*X = T, B*) runs over the extended part of the extended molecule:
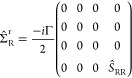
4a

and
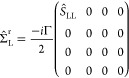
4bwith *Ŝ*_*XX*_ (X = L, R) being the left (L) or right (R) subspace of the overlap matrix (*Ŝ*) and Γ a numerical parameter. This self-energy model mimics the semi-infinite substrate environment effects in a finite region (the metallic part of the extended molecule) by a local absorption potential that gives a fair amount of broadening of the resonant peaks. This approach can be simplified building *H*_0_ + Σ^r^, using the eigenvectors of *H*_0._(38) To do this, we consider the set of the Löwdin^39,40^ orthonormalized |α̅⟩ and nonorthonormalized |α⟩ eigenvectors for *H*_0_. The relation between the eigenvectors |α̅⟩ and |α⟩ is given by |α̅⟩ = *Ŝ*^1/2^|α⟩ and, for the retarded self-energy operators, the relation is given by^40^ Σ̅_*X*_^r^ = *Ŝ*^–1/2^ Σ̂_*X*_^r^*Ŝ*^–1/2^, for *X* = L or R. Thus, we can define:
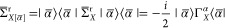
5a

and
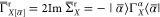
5bwith Γ_*X*_^α^ = ΓΩ_*X*_^α^ and .

It should be noted that, since in an orthonormal basis set |μ̅⟩ a molecular orbital (MO) |α̅⟩ can be written as a linear combination of atomic orbitals (LCAO) (|α̅⟩ = ∑_μ_*C̅*_μ_^α^|μ̅⟩), the term Ω_*X*_^α^ in eq 5b means the degree of localization of the α-MO in the left (*X* = L) or in the right (*X* = R) cluster of the extended molecule. In other words,eqs 5aand5b shows that for a specific eigenvalue (α), the level-broadening is dependent on the probability of finding the molecular orbital localized in the left and right gold cluster regions, implying that the main contribution for the self-energy will come from states with larger coefficients of the LCAO expansion of the wave function in these regions.

### 2.2 Model for the Coupling between the Contacts (*V*_LR_)

Since the left and right gold clusters are coupled with each other through a single molecule, the term *V̂* can be modeled by an effective potential using subspaces of the overlap matrix and the eigenvectors of the system, as we will show in next paragraphs.

**Figure 2:**
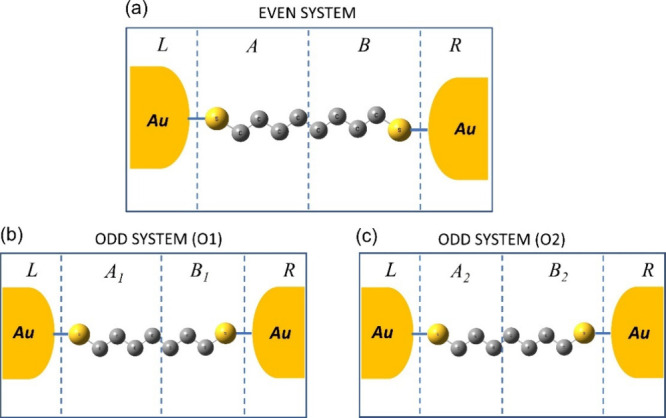
(a) Partition of the extended molecule with an even number of carbons in the chain into four spatial regions: A, B, L, and R. (b) One possible way to divide the extended molecule with an odd number of carbons in the chain. (c) Another possible way to divide the extended molecule with an odd number of carbons in the chain.

To model de potential *V*, we will consider four subspaces of the system labeled as L (left cluster), A (left side of the organic part), B (right part of the organic part), and R (right cluster), as shown in Figure 2a–c. It should be noted that (see Figure 2a–c) if for an even number of carbons in the chain, we can divide the system exactly in half (see Figure 2a) with the same number of carbons in regions A and B, for odd number of carbons, we have two possibilities, as depicted in Figure 2b,c. Thus, if for the even case, we define *V*_even_ as
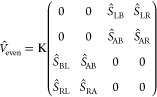
6

for the odd case, we can symmetrize averaging the two possibilities. Thus, we have
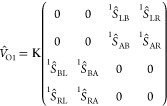
7a

or
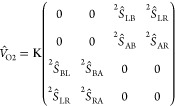
7b

and, finally
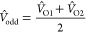
7c

It should be noted that each block in *V* indicates a possible path for an electron to traverse the system going from one contact to another. For example, block *S*_LR_ (or *S*_RL_) can be viewed as a direct link from one contact to another that occurs when the wave function is localized only at the clusters. On the other hand, block *S*_AB_ can be viewed as a transport from one contact to another, passing through the molecule, that occurs when the wave function is delocalized through all the system. The same reasoning can be applied for the other terms and, in what follows, using the eigenvectors of *H*_0_, considering the set of the Löwdin orthonormalized |α̅⟩ and the representation of this operator (*V*) in this base (|α̅⟩),^39,40^ i.e., *V̅* = *Ŝ*^–1/2^*V̂ Ŝ*^–1/2^. Similar for the self-energy, we can define:
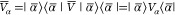
8where *V*_α_ = *K*Ω_*XY*_^α^, , with [*X*, *Y* = A, B, L, R and *X* ≠ *Y*], and *K is* a constant parameter that will be specified latter.

Finally, defining the partial Hamiltonian *H̅*_*XX*_ = |α̅⟩ (*E*_α_–*i* Γ_*X*_^α^)⟨α̅|, with X = L, R, and *E*_α_ being the energy of the molecular orbital labeled by α, the local density of states may be obtained by the usual relation^14,41^ π ρ̂_*X*_(*E*) = −Im *Ĝ*_*XX*r_(*E*), with *Ĝ*_*XX*r_ being the retarded Green′s function projected onto the subspace *X* (X = L, R), and the transmission, as defined in eq 2, can be explicitly written as
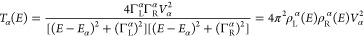
9

It should be noted that the transmission is defined for a given α-MO, i.e., *T*(*E*) → *T*_α_(*E*), with the total transmission being the sum *T*_Tot_(*E*) = ∑_α_*T*_α_(*E*) in the |α̅⟩-basis. In what follows, we will apply the model for a family of alkanes chains with odd and even numbers of carbons, adopting the set of parameters: Γ = 0.1 (see eqs 4a-4b), *K* = 1 (see eqs 6 and 7a-7c, and the Fermi level of the system as *E*_F_ = 5.53 (the gold Fermi energy), with all units in eV.

## 3 Results and Discussions

Once the electronic structure of the system is defined, we use our model in eq 9. To do this, we extract the parameters we need from quantum chemistry calculations to construct our model self-energy, as discussed in the previous section (see eqs 4-8). These calculations were carried out without the presence of an electric field since the field application does not significantly change the results.

**Figure 3:**
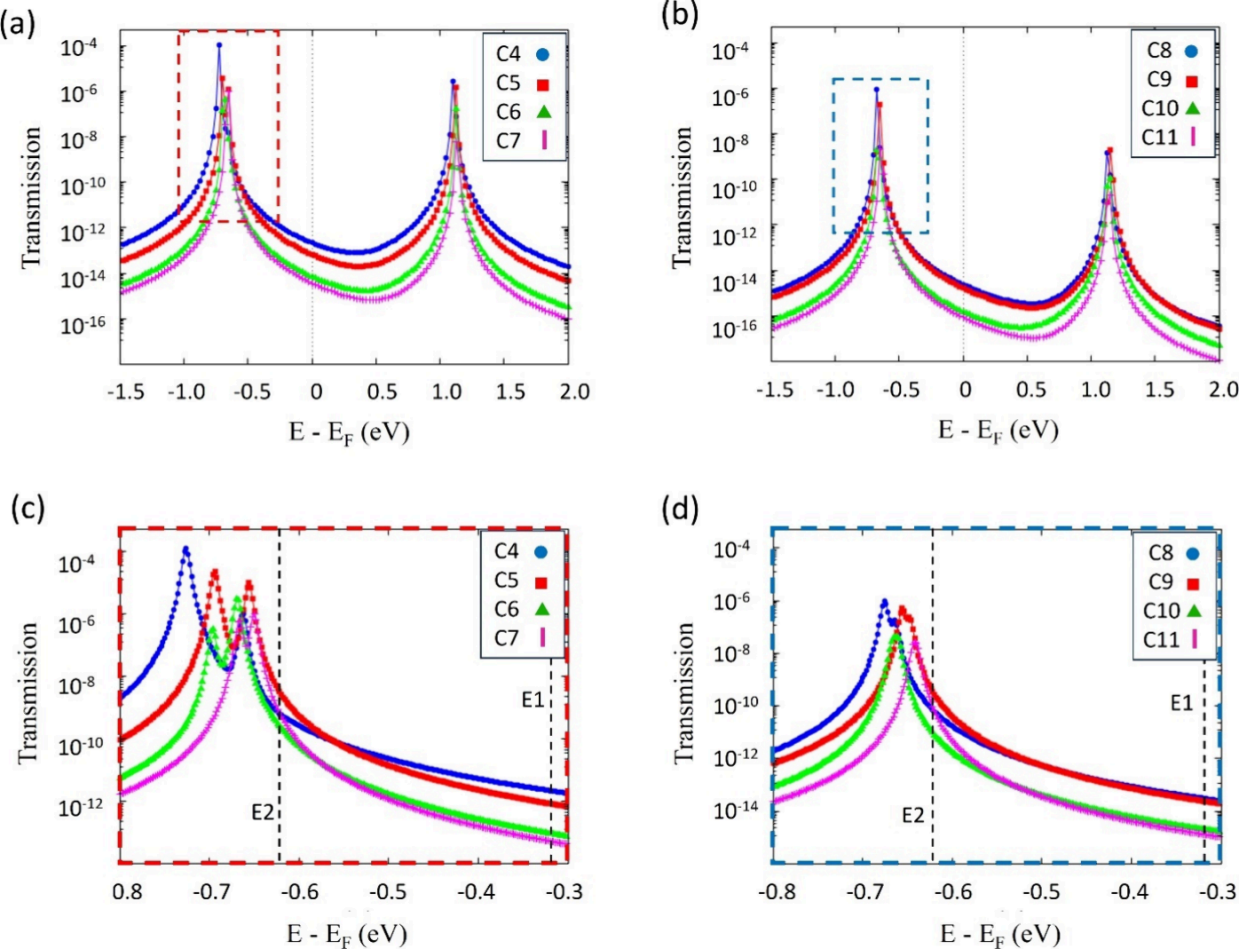
In (a) transmission functions of HOMO–1, HOMO, LUMO, and LUMO+1 of C_4_, C_5_, C_6_, and C_7_. In (b) transmission functions of HOMO–1, HOMO, LUMO, and LUMO+1, for C_8_, C_9_, C_10_, and C_11_. (c) Ampliations of the MOs peaks for systems C_4_, C_5_, C_6_, and C_7_. In (d) ampliation for systems C_8_, C_9_, C_10_, and C_11_ (d). The energies *E*_1_ and *E*_2_ are indicated by vertical dotted lines.

In Figure 3a,b, the transmission function is presented as a function of the shifted energy (*E–E*_F_) for C_4_ to C_7_ (Figure 3a) and for C_8_ to C_11_ (Figure 3b). It can be seen that, for all systems, the Fermi energy of gold is closer to the HOMO than to the LUMO, thus making HOMO and HOMO*–*1 the most important conduction channels. The fact that the HOMO is closer to the Fermi level has been discussed in the literature in the case of alkanes.^12,42^ It is also quite noticeable that as the size of the molecular chain increases, the degeneration of both orbitals (HOMO and HOMO*–*1) becomes increasingly more pronounced. This fact is shown in Figure 3c,d, where a zoom of the region delimited by dotted lines in Figure 3a,b shows two distinct peaks corresponding to the energy values of HOMO*–*1 and HOMO for small chains (see Figure 3c). In the case of longer chains, a pronounced approximation of both peaks is observed (Figure 3d). It should be noted that not only does the energy gap between HOMO and HOMO*–*1 decrease but also the height of the transmission peaks also changes as a function of the size of the chain. Thus, as shown in Figure 3a–d, increasing the number of carbons in the chain decreases the height of the peaks. This behavior is related to the weakening of the coupling between the molecule and the electrodes with the increase in the length of the molecular chain. This is reported in many experimental^17,43,44^ and theoretical works.^19,45^

**Figure 4:**
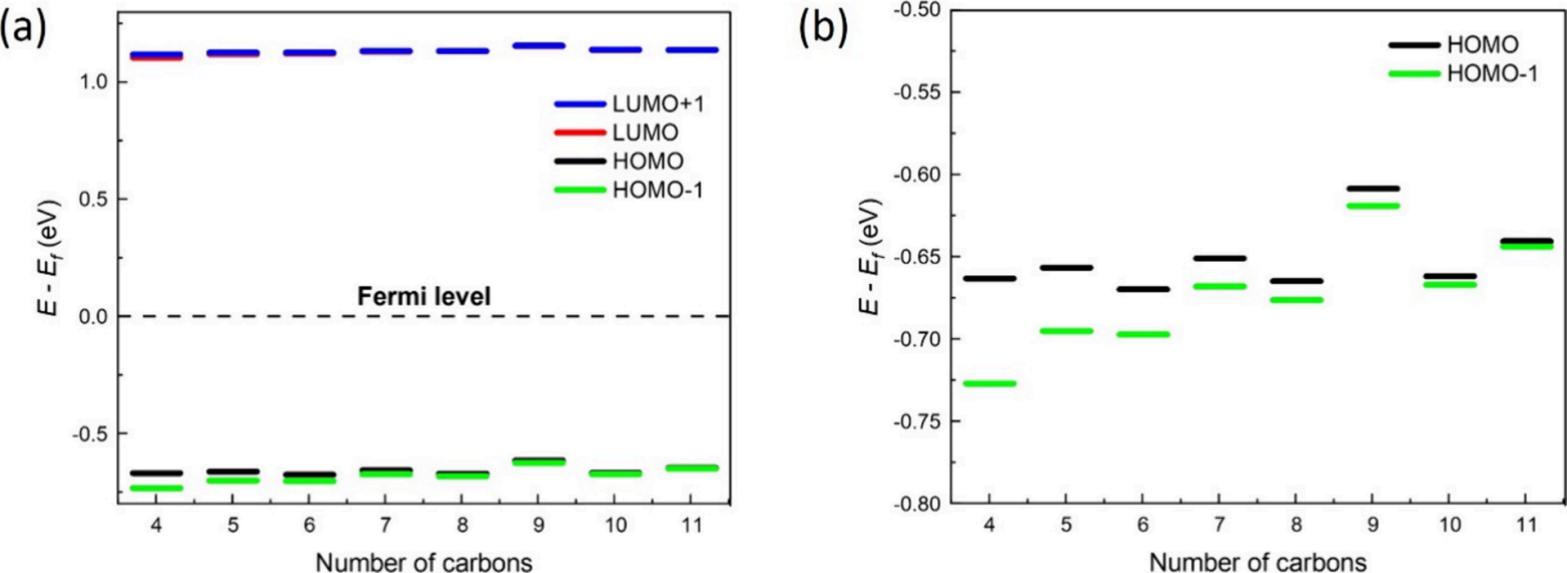
(a) Energy levels of the frontier orbitals in relation with de Fermi level. (b) A zoom in (a) shows the alternation in the energy levels values for HOMO and HOMO–1.

Another interesting behavior of these systems is the shift in the eigenvalues of HOMO*–*1 and HOMO, as shown in Figure 4a,b. As shown above, the decrease in the gap between the eigenvalues of HOMO and HOMO–1 is related to the size of the molecule. However, the shift observed more clearly in Figure 4b points being a parity effect: for systems with odd number of carbons in the chain, the HOMO’s eigenvalue is slightly higher than for even number of carbons in the chain. This fact can also be viewed in Figure 3c, where the HOMO peaks of the transmission function for C_5_ and C_7_ systems are shifted to the right (thus, nearest *E–E*_F_ = 0) when compared with the HOMO’s peaks for C_4_ and C_6_. It can also be noted in Figure 3d that the HOMO peaks of the transmission function of C_9_ and C_11_ are shifted to the right compared to those of C_8_ and C_10_. The odd–even effect can be appreciated when analyzing the transmission function and is more easily corroborated by observing Figure 4d.

**Figure 5:**
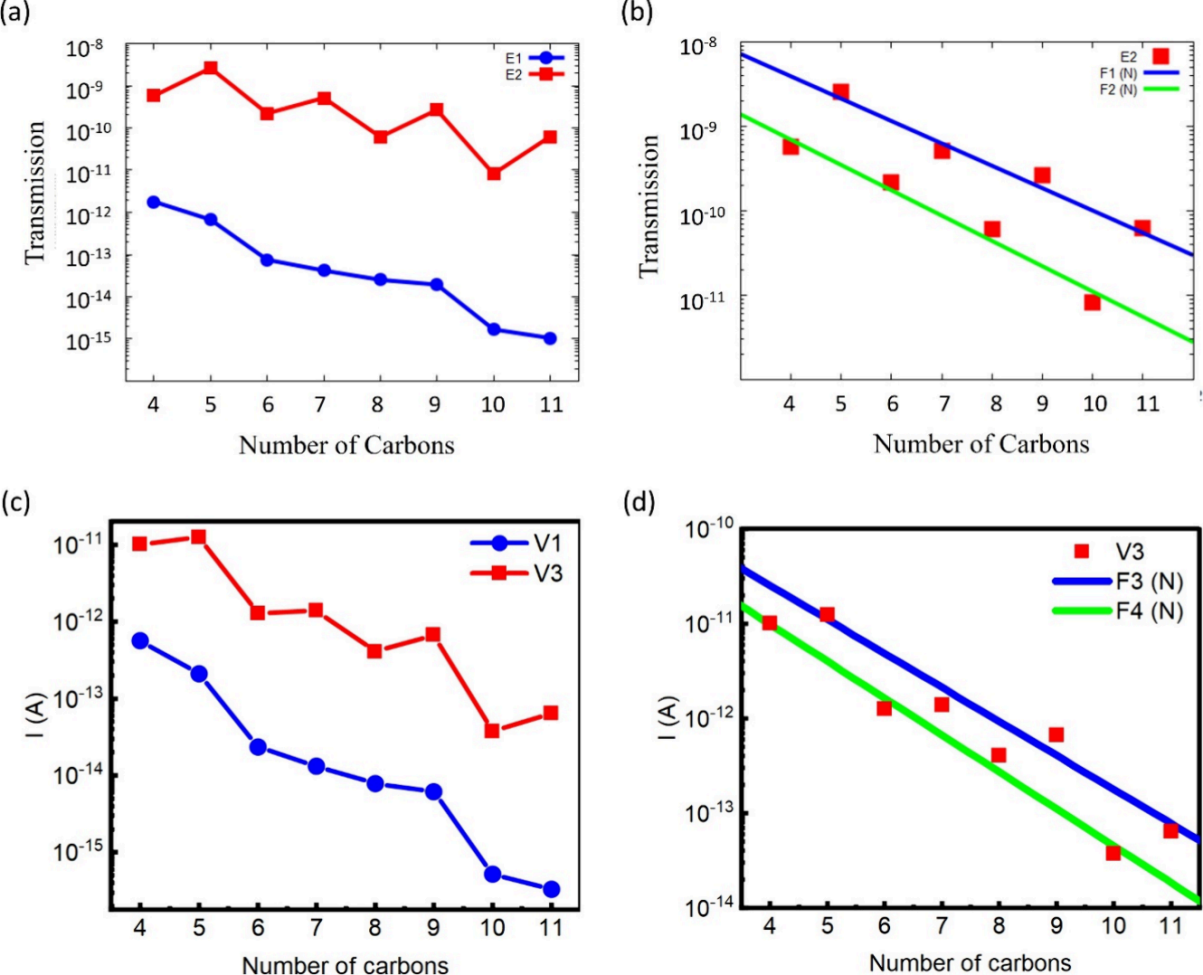
Values of the transmission function at energies *E*_1_ and *E*_2_ versus the number of carbon atoms (a). Linear fit of the transmission function values on the *E*_2_ energy for odd and even alkanes (b). Values of the electric current at voltages *V*_1_ and *V*_3_ vs the number of carbon atoms (c). Linear fit of the transmission function values on the *V*_3_ voltage for odd and even alkanes (d).

To better explore the odd–even effect showed above, the transmission function in the off-resonant and near-resonant regions related with the HOMO eigenvalue was examined. For this, it was considered to analyze the transmission function at the energy values *E*_1_ (*E*_1_ = *–*0.32 eV) and *E*_2_ (*E*_2_ = *–*0.63 eV). It should be noted that *E*_1_ is designated as the off-resonance energy because it is far from the energy at which HOMO and HOMO–1 are located. On the other hand, *E*_1_ is the energy value near the energy where the peaks of transmission function of the HOMO and HOMO–1 are located (see Figure 3c,d). The transmission functions corresponding to the energy values *E*_1_ and *E*_2_ for each molecule are shown in Figure 5a. It should be noted that, depending on the energy position, a distinct behavior of the transmission function can be obtained: an almost monotonically (*E* = *E*_1_) or an oscillatory (*E* = *E*_2_) behavior. In the first case (*E* = *E*_1_), the shift in energy due to the parity does not almost alter the decreasing behavior of the transmission function of the molecules. As a consequence, no odd–even effect appears far from resonance, and there was a monotonic decreasing dependence of the transmission with the length of the molecule. These results are experimentally corroborated by Reed and co-workers^46^ in the case of coherent, off-resonant tunneling. Reed et al. showed that the conductance of alkanedithiol molecules shows an exponential decrease with the molecular length in the off-resonance regime, i.e., far from HOMO or LUMO transmission peaks.

A completely distinct behavior can be found near resonance (*E* = *E*_2_), as shown in Figure 5a. In this case, there is an oscillatory behavior, and the reason is related to the shift of the eigenvalues of HOMO and HOMO–1 (Figures 3c,d and 4b). Furthermore, the values of the odd and even alkanes can form two different sets described by an exponential decay function (*F*(*n*) ∼ exp(−β·*n*)), each one with its own decay constant:^15^ β_even_ = 0.69 and β_odd_ = 0.61. This fact can be seen in Figure 5b where the linear fits for a semilog plot of the transmission values for the odd and even alkanes are shown. The behavior of the function on *E*_2_ explains the oscillatory behavior of the electrical current.

The behavior of the current at 0.8, 1.0, and 1.2 V was also studied using eq 1. Table 1 shows the current values at the aforementioned voltage values for all of the systems studied. Figure 5c shows the current values at 0.8 V (nonresonant) and 1.2 V (near-resonant). It is easy to see the similarity with Figure 5a: in the case of voltages within the nonresonant range (0.8 V), an almost monotonic decrease is noted. In the case of voltages in the resonant range (1.2 V), an oscillatory behavior is perceived. As with the transmission function near the resonance, the current values can form two sets, one for the even molecules and another for the odd molecules (see Figure 5d). If we use the exponential function to fit the curves of the two sets of current values, we find that β_even_ = 0.89 and β_odd_ = 0.82.

**Table 1:** Values of Electrical Current *I* (in Amperes) for All Systems Studied, for Some Values of Voltage (*V*_N_) in Voltz

system	*V*_1_ = 0.8 V	*V*_2_ = 1.0 V	*V*_3_ = 1.2 V
C_4_	*I =* 5.62 × 10^–13^	*I =* 1.71 × 10^–12^	*I =* 1.02 × 10^–11^
C_5_	*I =* 2.10 × 10^–13^	*I =* 8.10 × 10^–13^	*I =* 1.25 × 10^–11^
C_6_	*I =* 2.35 × 10^–14^	*I =* 9.45 × 10^–14^	*I =* 1.27 × 10^–12^
C_7_	*I =* 1.32 × 10^–14^	*I =* 5.90 × 10^–14^	*I =* 1.39 × 10^–12^
C_8_	*I =* 7.83 × 10^–15^	*I =* 3.10 × 10^–14^	*I =* 4.10 × 10^–13^
C_9_	*I =* 6.11 × 10^–15^	*I =* 2.79 × 10^–14^	*I =* 6.73 × 10^–13^
C_10_	*I =* 5.23 × 10^–16^	*I =* 2.21 × 10^–15^	*I =* 3.77 × 10^–14^
C_11_	*I =* 3.29 × 10^–16^	*I =* 1.65 × 10^–15^	*I =* 6.45 × 10^–14^

It should be noted that both the oscillatory behaviors (of the current and that of the transmission function) are reported in the literature.^18,21^ For example, Wei and Wang found that this oscillation of the current with the number of carbons in the alkane chain has an odd–even effect on the rectification ratio in the context of studying a molecular rectifier, where an asymmetric single molecule is used. In the case of experimental works, the odd–even effect on the current density of alkane SAMs has been studied extensively.^15,47,48^ More recently, Amara et al. studied the odd–even effect on other properties such as capacitance, dielectric constant, and hydrophobic surface.^49^ However, experimental work was not reported at the single-molecule level. It is important to make clear that the molecules studied in this paper are symmetrical, and the oscillatory behavior of the current that we report was present in symmetrical molecules as well. This, to the best of our knowledge, has not been reported previously.

**Figure 6:**
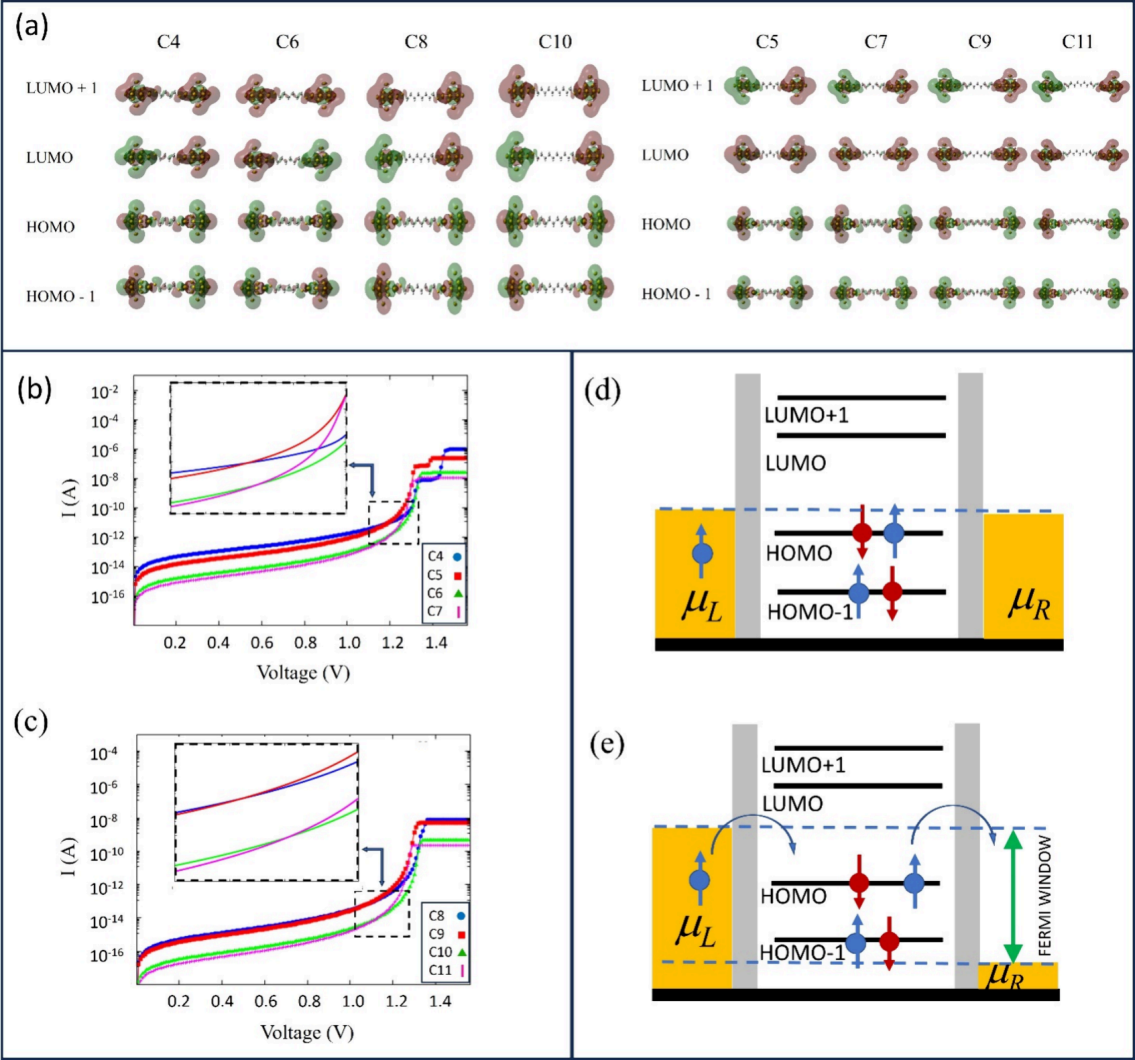
(a) LUMO+1, LUMO, HOMO, and HOMO–1 electron density map (isovalue = 0.005) of the even (left) and odd (right) alkanes. In (b) *I*–*V* curves of C_4_, C_5_, C_6_, and C_7_ and in (c) for C_8_, C_9_, C_10_, and C_11_. The inset shows the inversion of the electric current near the resonant region. In (d), a schematic representation of the system with *V* = 0 and in (e) for *V* ∼ 1 V when HOMO and HOMO–1 are inside the Fermi window.

The spatial localization of some frontier orbitals (HOMO–1, HOMO, LUMO, and LUMO+1) corresponding to the peaks in the transmission function is shown in Figure 6a. As the molecular junction is symmetric, the localization is also symmetric for all orbitals. Furthermore, it can be observed that the electron density is more localized at the gold clusters. However, in the case of occupied orbitals (HOMO and HOMO–1), we have some delocalization through the system when compared with the unoccupied MOs (LUMO and LUMO+1), showing that the occupied orbitals have some electronic density distributed in the backbone of the molecule. This delocalization is more evident for the smaller systems and tends to reduce when the size of the system increases. This fact is of great importance since the nature of the electronic density of each MO is relevant for our model. The key parameters in the transmission function (see eq 2) are the coupling strength at the middle point of the system (see eqs 6 and 7a-7c) and the localization of the wave function in the extended part of the extended molecule (see eqs 4aand4b). This fact explains why the peaks of the transmission function are higher for occupied MOs (HOMO and HOMO–1) and decrease (for all MOs) with the size of the system according to our model.

After the previous analysis of specific voltage values, the behavior of the electric current was examined in a wider voltage range. We have plotted the curves from C_4_ (Figure 6b) to C_7_ and from C_8_ to C_11_ (Figure 6c). In both figures, it is noted that the smallest molecules have the highest current values, as expected. As showed in Figure 6b,c, for voltage values lower than 1.0 V, all electrical current curves had higher values for smaller molecules for all systems (C_4_–C_11_) since it was in the off-resonant region. However, an alteration of the previous order in the electrical current occurs between the pairs of systems C_*N*_ and C_*N*+1_ (*N* = 4, 6, 8 and 10) when the Fermi window reaches the occupied frontier orbitals (especially the HOMO), as shown in Figure 6d,e and in their insets. As already discussed above, the eigenvalues of the odd systems are shifted, and the transmission function for these systems rises before the transmission function of the even systems. Thus, an important change is perceived when the voltage is around 1.2 V, and the order in the off-resonant region of the current values has completely altered. In the case of Figure 6b, the current on the C_5_ and C_7_ molecules exceeds the current on C_4_ and C_6_, respectively, as shown in the inset of Figure 6b and in Table 1. The same is shown in Figure 6c (see the inset) with molecules C_9_ and C_11_, exceeding C_8_ and C_10_, respectively, in their current values (Table 1).

Going further in the voltage, the higher values in the transmission function for small systems start to prevail in the calculation of the electric current. Another alteration occurs in the region between 1.3 and 1.4 V. The electric current for small systems was larger again. At this point, plateaus are observed until new MOs (HOMO–*N*, *N* > 2, for example) start to contribute to the increase in the electrical current. We stress here that, in the off-resonant regime (far from the transmission peaks), direct tunneling contributions are present, but their magnitudes are several orders (∼10^–3^) smaller than the resonant contributions.

## 4 Conclusions

In this work, the transmission function and the electrical current for alkane molecules of different sizes were calculated. For this, we construct eight extended molecules composed by alkanes attached to two small gold clusters via thiol groups, with the number of carbons varying from *n* = 4 to *n* = 11 and thus, odd–even effects can be studied. Analysis of the results revealed that the electrical response does not decay exponentially with the size of the molecule but depends on the parity of the number of carbons in the chain and whether the voltage where the conductance is measured is close to or far from a resonance with some frontier molecular orbitals. Far from resonance, despite the results not showing an exponential decay, the size of the system is still the most relevant variable: greater the size of the chain, smaller the observed conductance. This behavior changes when the voltage is increased and the resonance with the frontier orbitals is reached, especially for HOMO. In this case, we reported an odd–even effect: an oscillatory behavior of the transmission function. This effect is due to the alternation of the energy levels of the frontier orbitals. Thus, near resonance, this causes odd-numbered molecules to have a greater response than even-numbered molecules, even though they are larger. To the best of our knowledge, a first-principles study together with the CAPs technique of the odd–even effect in symmetric systems composed by alkanes covalently coupled to electrodes has not been reported yet.
